# Opportunities for Earlier Diagnosis and Treatment of Cardiac Amyloidosis

**DOI:** 10.14797/mdcvj.1163

**Published:** 2022-12-06

**Authors:** Trejeeve Martyn, Andres Carmona Rubio, Jerry D. Estep, Mazen Hanna

**Affiliations:** 1 Robert and Suzanne Tomsich Department of Cardiovascular Medicine, George and Linda Kaufman Center for Heart Failure and Recovery, Cleveland Clinic, Cleveland, Ohio, US; 2 Amyloidosis Center, Cleveland Clinic, Cleveland, Ohio, US; 3 Department of Cardiovascular Medicine, Cleveland Clinic Florida, Weston, Florida, US

**Keywords:** amyloidosis, transthyretin amyloid, screening, cardiomyopathy, early diagnosis

## Abstract

Despite the rapid expansion of noninvasive (nonbiopsy) diagnosis, contemporary patients with cardiac amyloidosis too often present with advanced features of disease, such as diminished quality of life, elevated natriuretic peptides, and advanced heart failure. Therapeutics for transthyretin cardiomyopathy (ATTR-CM) are most effective when administered before significant symptoms of cardiac dysfunction manifest, making early identification of affected individuals of paramount importance. Community engagement and ensuring that a broad range of clinicians have working knowledge of how to screen for ATTR-CM in everyday practice will be an important step in moving disease identification further upstream. However, reliance on the appropriate and timely diagnosis by individual clinicians may continue to underperform. This review highlights how targeted screening of special populations may facilitate earlier diagnosis. Systems of care that operationalize screening of high-risk subpopulations and prospective validation of novel approaches to ATTR-CM identification are needed.

## Background

Cardiac amyloidosis (CA) is characterized by the extracellular deposition of amyloid fibrils in the heart with the distinctive histological property of green birefringence when viewed under cross polarized light microscopy after staining with Congo red.^1,2^ There are two main types of CA, namely transthyretin (TTR) cardiomyopathy (ATTR-CM) and light chain (AL) cardiomyopathy (AL-CM). In ATTR-CM, the native tetrameric form of TTR, produced mainly in the liver, pathologically dissociates to form amyloid fibrils that deposit in the myocardium, nerves, and soft tissues.^3^ AL-CM occurs when an abnormal clone of plasma cells produces a light chain that is prone to misfolding and forming amyloid fibrils that deposit in the myocardium, kidneys, GI tract, liver, nerves, and soft tissues. In both instances, the myocardium becomes progressively thickened, causing heart failure, conduction disease, and arrhythmias.^3,4,5^

ATTR amyloidosis can occur as an acquired disease of aging—called “wild type” (ATTRwt)—which is most commonly found in older White males but has no known genetic cause. It also can be due to an autosomal dominant inherited pathogenic variant in the *TTR* gene (ATTRv) with more than 150 variants described to date; some present predominantly with cardiomyopathy, others with peripheral and/or autonomic neuropathy, and most with a mixed phenotype. The most common variant in the United States is the valine to isoleucine substitution at amino acid 122 (V122I or p.V142I), of which 3% to 4% of African Americans are heterozygote carriers, putting them at risk for the phenotypic expression of late onset ATTR-CM.^5,6^ Median survival after diagnosis in untreated patients is poor: 2.5 years for ATTRv-CM caused by the V122I variant and 3.6 years for ATTRwt-CM.^7,8,9,10^

**Figure 1:**
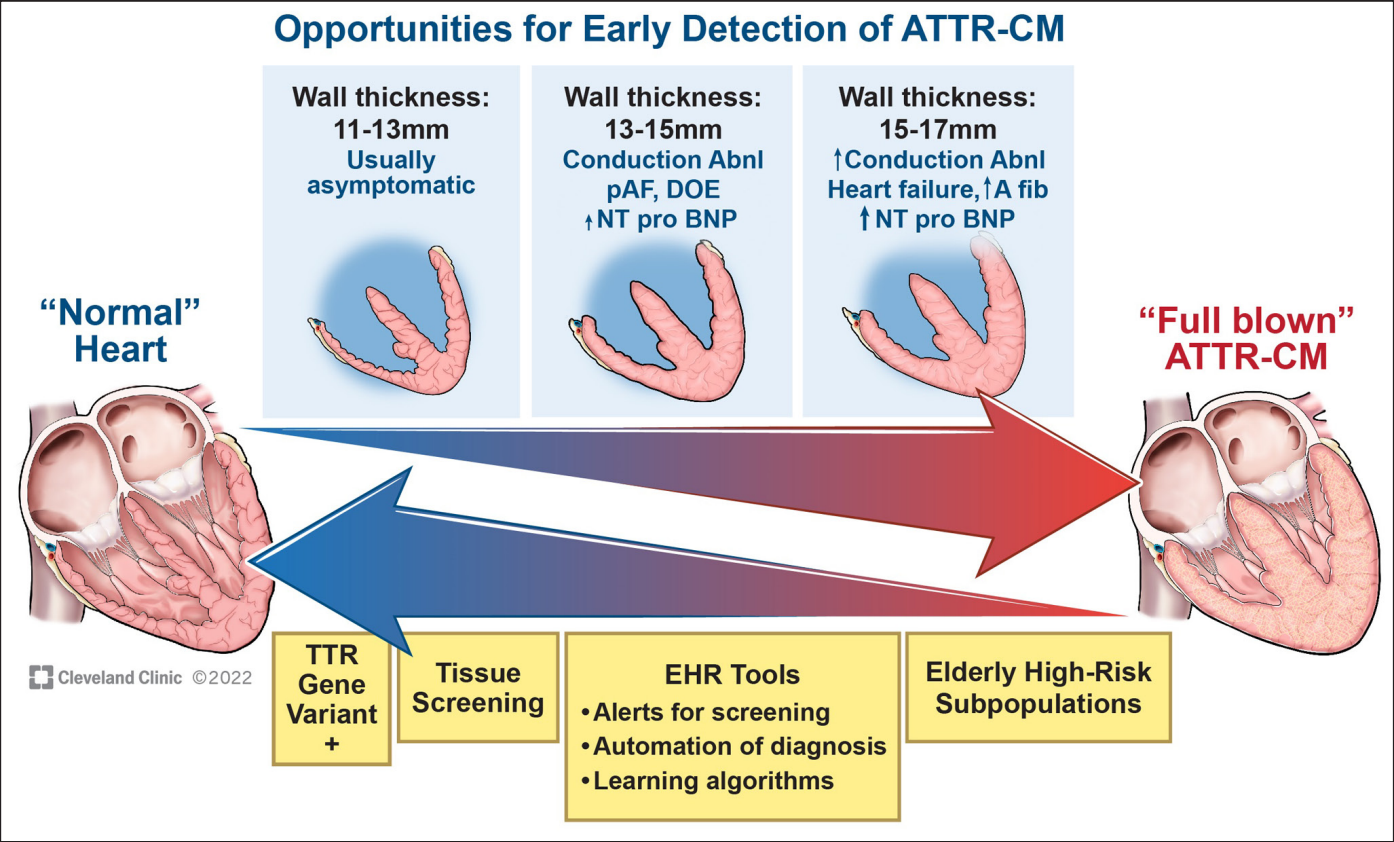
Top arrow represents a conceptual diagram of progression across time from a normal heart to advanced transthyretin (TTR) amyloid cardiomyopathy (ATTR-CM). Bottom arrow depicts different opportunities during the disease process for earlier detection of ATTR-CM. abnl: abnormalities; pAF: paroxysmal atrial fibrillation; DOE: dyspnea on exertion; NT-proBNP: N-terminal pro-brain natriuretic peptide; EHR: electronic health record *Note*: this diagram is meant to be conceptual, and manifestations of disease may vary across the spectrum of wall thickness. * Conduction abnormalities include first-degree heart block, right bundle branch block (BBB), left BBB, intraventricular conduction delay, complete heart block. ** High-risk subpopulations: specialized heart failure clinics, inpatient heart failure admissions, transcatheter aortic valve replacement, Black patients.

Recent data suggest that CA has been an underrecognized cause of cardiac disease.^11,12,13,14^ Although awareness has increased, accurate and timely diagnosis remains suboptimal. This may be due in part to late recognition by clinicians as well as uneven access to noninvasive multimodality imaging tools and/or endomyocardial biopsy needed for the diagnostic evaluation. Furthermore, the attribution of the presenting symptoms and signs to aging, hypertension, hypertrophic cardiomyopathy, and other causes of heart failure and diastolic dysfunction contribute to missed and delayed diagnosis.^4,5,6,7^ Efforts to move the therapeutic window further upstream from the decline in quality of life, worsening renal function, elevation of natriuretic peptides, and shortened life expectancy associated with advancing ATTR-CM would have tremendous benefit to patients (Figure 1).^15^

Given the morbidity and diminishing treatment benefit associated with late-presenting disease, the goal of this review is to highlight opportunities to facilitate the earlier identification of CA, with a focus on ATTR-CM. The features, evaluation, and approach to treatment of AL amyloidosis is reviewed elsewhere, including a recent issue of this journal.^5,16^

This review sets out to (1) highlight patient characteristics as well as multimodality imaging, electrocardiographic, and biomarker findings that should lead to a heightened clinical suspicion for ATTR-CM; (2) briefly touch on the differential diagnosis and diagnostic workup of ATTR-CM; (3) underscore the ongoing problem of missed or late diagnosis in the context of expanding therapeutic options for ATTR-CM; and (4) highlight opportunities for earlier diagnosis of ATTR-CM, including tissue screening during surgical procedures, noninvasive screening of high-risk subpopulations, electronic health record (EHR)-based strategies to bolster proactive diagnosis, and serial evaluation of asymptomatic carriers of *TTR* gene variants.

## Part I: Developing Clinical Suspicion for ATTR-CM

It is important to recognize patient demographics, including sex, race, and age in the context of the clinical picture. Patients typically present with symptoms of heart failure but also can present with symptoms of atrial fibrillation and/or conduction disease.^1^ A history of hypertension with now normal blood pressure or requiring a decrease in the medication dose is a potential clue to the diagnosis of ATTR-CM.^1,17^ The evaluation of a patient with heart failure, arrhythmia, or conduction disease should begin with a review of the echocardiogram (echo) and electrocardiogram (ECG) in addition to a comprehensive history.

### Echocardiogram

Increased wall thickness is a fundamental consequence of the development of ATTR-CM. Given that the upper limit of normal left ventricular (LV) wall thickness is 10 mm for males and 9 mm for females, it has been traditionally stated that a wall thickness of ≥ 12 mm in the absence of another clear etiology, such as uncontrolled hypertension or aortic stenosis, should prompt a suspicion for infiltrative heart disease.^18^ Witteles et al. put forth a schema recommending that CA be considered when a wall thickness of ≥ 14 mm is seen by echo. While this degree of wall thickening may be more specific, this cutoff may be less sensitive for the earlier diagnosis of ATTR-CM.^4^ Of note, the average wall thickness at diagnosis in most series and in the ATTR-ACT trial is 16 mm to 18 mm, well beyond that of what we are trying to achieve for earlier diagnosis.^4,19,20^ Furthermore, the presence of hypertension and/or aortic stenosis in a thickened ventricle does not exclude the coexistence of ATTR-CM. Other findings on echo are a nondilated left ventricle, mitral and tricuspid valve thickening, and biatrial dilatation.^3,21^ Additionally, while ATTR-CM is often considered a disease of heart failure with preserved ejection fraction (HFpEF), recent data from our institutional registry indicates that impaired LV function is an overlooked phenotype in patients with both ATTRwt-CM and ATTRv-CM. Nearly half of the patients presented with impaired LVEF < 50%, with more than a quarter of them presenting with HF with reduced EF (LVEF ≤ 40%).^22^ Finally, leveraging the application of longitudinal strain imaging in any patient with thickened LV walls can help differentiate CA from other causes of myocardial thickening. Diminished longitudinal strain with apical sparing is a pattern that is well described in advanced CA.^23^

### Electrocardiogram

The echo evaluation should always take place with the concomitant review of the ECG. The discordance between LV wall thickness and QRS voltage has been noted as a finding to raise clinical suspicion of CA. Normal or even increased voltage should not provide reassurance when other concerning history, symptoms, imaging, or biomarker findings are suggestive of CA.^2,24^ Indeed, in a cohort of 400 patients with ATTR-CM, low voltage was found in only 33%.^18^ Atrial fibrillation is a common rhythm disorder in elderly patients, but it has a very high prevalence in patients with ATTR-CM, particularly wild type.^25^ Therefore in an older patient with atrial fibrillation and a thickened LV wall and/or other red flags such as carpal tunnel syndrome, the index of suspicion for ATTR-CM should be heightened.^17,26^ Additionally, in the right clinical context, conduction disease on ECG such as PR prolongation, increased QRS duration with intraventricular conduction delay, left or right bundle branch block, or prior pacemaker are important clues to strengthen suspicion.^27,28^

### History and Symptomology

**Figure 2:**
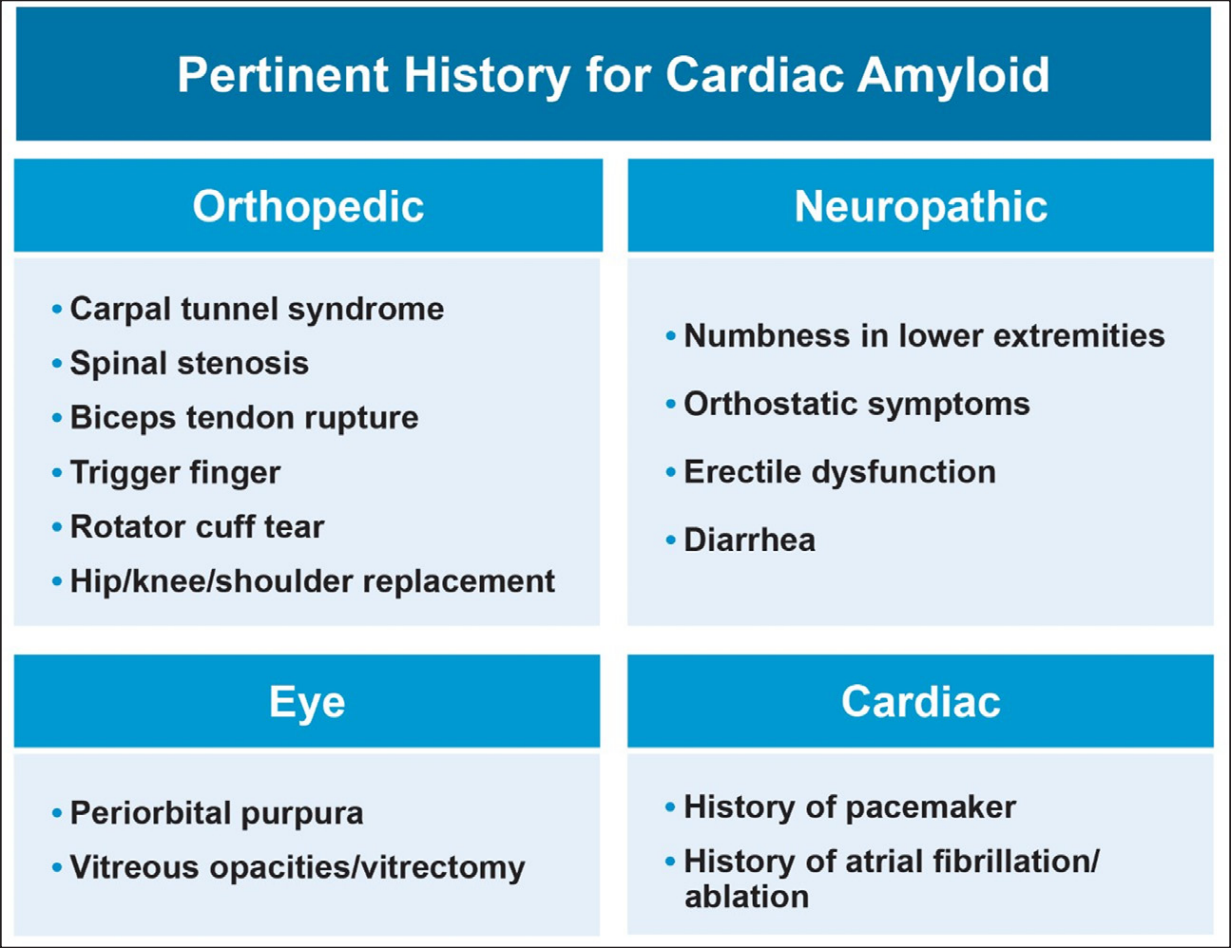
Past medical/surgical history and symptoms that can serve as red flags to raise clinical suspicion for cardiac amyloidosis.

A thorough history is of paramount importance and should include questions regarding noncardiac symptoms or previous surgeries. Because ATTR amyloid deposits in the soft tissues, ligaments, joints and tendons, a comprehensive orthopedic history should be taken (Figure 2).^3^ Several common clinical conditions, including carpal tunnel syndrome, lumbar spinal stenosis, trigger finger, and bicep tendon rupture, are documented extracardiac manifestations of ATTRwt-CA.^29,30,31,32,33,34^ Bilateral carpal tunnel syndrome and lumbar spinal stenosis can precede the cardiac diagnosis by several years. A recent study found that bilateral carpal tunnel syndrome was present in approximately 50% of individuals with ATTRwt-CM in the 5 to 7 years preceding the diagnosis.^35^

Additionally, asking questions pertaining to peripheral and autonomic neuropathy symptoms (ie, erectile dysfunction, orthostasis, numbness and/or weakness in the distal extremities, and diarrhea) can signal systemic amyloidosis and is more commonly seen in ATTRv-CM. A comprehensive family history is of particular importance due to the inherited forms of transthyretin amyloidosis.^7^

### Biomarkers

Both serum troponin and natriuretic peptide levels are often elevated in cases of clinically apparent CA.^3,27^ Unfortunately, by the time these biomarkers are substantially elevated, the disease is typically more advanced.^9,10^ An elevated troponin in the absence of an acute coronary syndrome in a patient with thickened LV walls can be a clue to CA.

### Cardiac MRI

Cardiac magnetic resonance imaging (CMR) is an extremely helpful tool to aid in the diagnosis of CA by helping to differentiate it from other causes of increased LV wall thickness. There are characteristic imaging findings regarding the pattern of late gadolinium enhancement (LGE) often showing a more diffuse subendocardial or transmural pattern as well as difficulty nulling the myocardium.^1,36^ Ultimately, neither a “negative” CMR in the context of a high clinical suspicion nor a characteristic CMR for CA obviates the need for confirmatory testing.

### Differential Diagnosis and Pre-test Risk

The differential diagnosis of ATTR-CM includes hypertensive heart disease (particularly when combined with chronic kidney disease), hypertrophic cardiomyopathy, Fabry’s disease, and others. The combination of biochemical (hs-cTnT), ECG (wide QRS), and structural (LV posterior wall thickness) findings were highly predictive components of a pre-test probability risk score for patients with suspected ATTR-CM derived from a Japanese cohort undergoing ^99m^Tc-PYP scintigraphy.^37^ Recently, a simple clinical risk score including readily available demographic and echo data was validated in a largely White group of HFpEF patients.^38^ The implementation of pre-test risk scores has the potential to guide clinicians with more limited clinical experience and can be incorporated into EHR-based alerting. Once there is clinical suspicion of CA, a comprehensive approach to confirmatory testing should be undertaken.^36^

## Part II: Diagnostic Workup

The appropriate steps for the diagnostic workup of CA has been well described, with published algorithms having in common the crucial need to rule out AL amyloidosis with simple laboratory testing and the pursuit of technetium-based cardiac scintigraphy.^1,36^ The appropriate tests to rule out AL amyloidosis are a serum free light chain assay and immunofixation of the serum and urine. The nuances of cardiac scintigraphy and the optimal approach to accurate imaging and incorporation of single photon emitted computed tomography (SPECT), and preferably SPECT/CT when available, are included in the American Society of Nuclear Cardiology (ASNC) guidelines.^36,39,40^ It is important to note that AL-CM can cause significant uptake on ^99m^Tc-PYP scan.^36^ False positives may occur due to blood pool in the LV cavity as opposed to true myocardial uptake, which needs to be differentiated by SPECT imaging. Finally, endomyocardial biopsy should be pursued when there is conflicting data to confirm amyloid deposits with accurate typing by immunohistochemistry (only with experienced pathologists) or mass spectrometry.

## Part III: Expanding Therapeutics and the Ongoing Problem of Late Diagnosis

A nuanced approach to earlier diagnosis of ATTR-CM is no longer an academic exercise since therapeutic options have expanded beyond cardiac and liver transplantation. The clinical use of the transthyretin stabilizer (tafamidis), the first ATTR-CM treatment approved by the United States Food and Drug Administration (FDA), has proven benefits with regard to mortality, hospitalization, and quality of life in both ATTRwt-CM and ATTRv-CM.^19,41^ Two TTR silencing drugs that significantly reduce the production of TTR by the liver—patisiran and inotersen—are FDA approved for the treatment of neuropathy in ATTRv, with clinical trials of silencer therapy in ATTR-CM either in progress or recently completed.^42,43^

The top-line announcement from APOLLO-B, a phase 3 double-blind placebo-controlled study examining the impact of patisiran on the primary end point of change in 6-minute walk distance at 12 months, showed benefit in patients with ATTR-CM. The full manuscript detailing the results is not available at the time of this review. Additionally, CRISPR-Cas9 in vivo gene editing for the treatment of TTR-related polyneuropathy has proven safe and effective at reducing TTR protein concentrations through the targeted knockout of the *TTR* gene with a single infusion of NTLA-2001 in six patients with ATTRv.^44^ A trial in patients with ATTR-CM is in the planning phases.

### Late Presentation and Missed Diagnosis

A longitudinal registry study from 2000 to 2017 catalogues the substantial diagnostic delay, high use of inpatient hospital services, and persistent diminishment of quality of life in the period prior to confirmatory diagnosis of both ATTRwt- and ATTRv-CM. Strikingly, the diagnosis of ATTRwt-CM was delayed > 4 years after initial presentation of cardiac disease in 42% of cases.^8^ A staging system built on natriuretic peptide and renal function thresholds developed by the National Amyloidosis Center (NAC)—with stage I defined as N-terminal pro-brain natriuretic peptide (NT-proBNP) ≤ 3,000 pg/mL and estimated glomerular filtration rate (eGFR) ≥ 45 mL/min/1.73 m^2^, stage III defined as NT-proBNP > 3,000 pg/mL and eGFR < 45 mL/min/1.73 m^2^, and the remainder of patients at stage II—has significant prognostic value with divergent survival across the three stages.^9^

**Figure 3:**
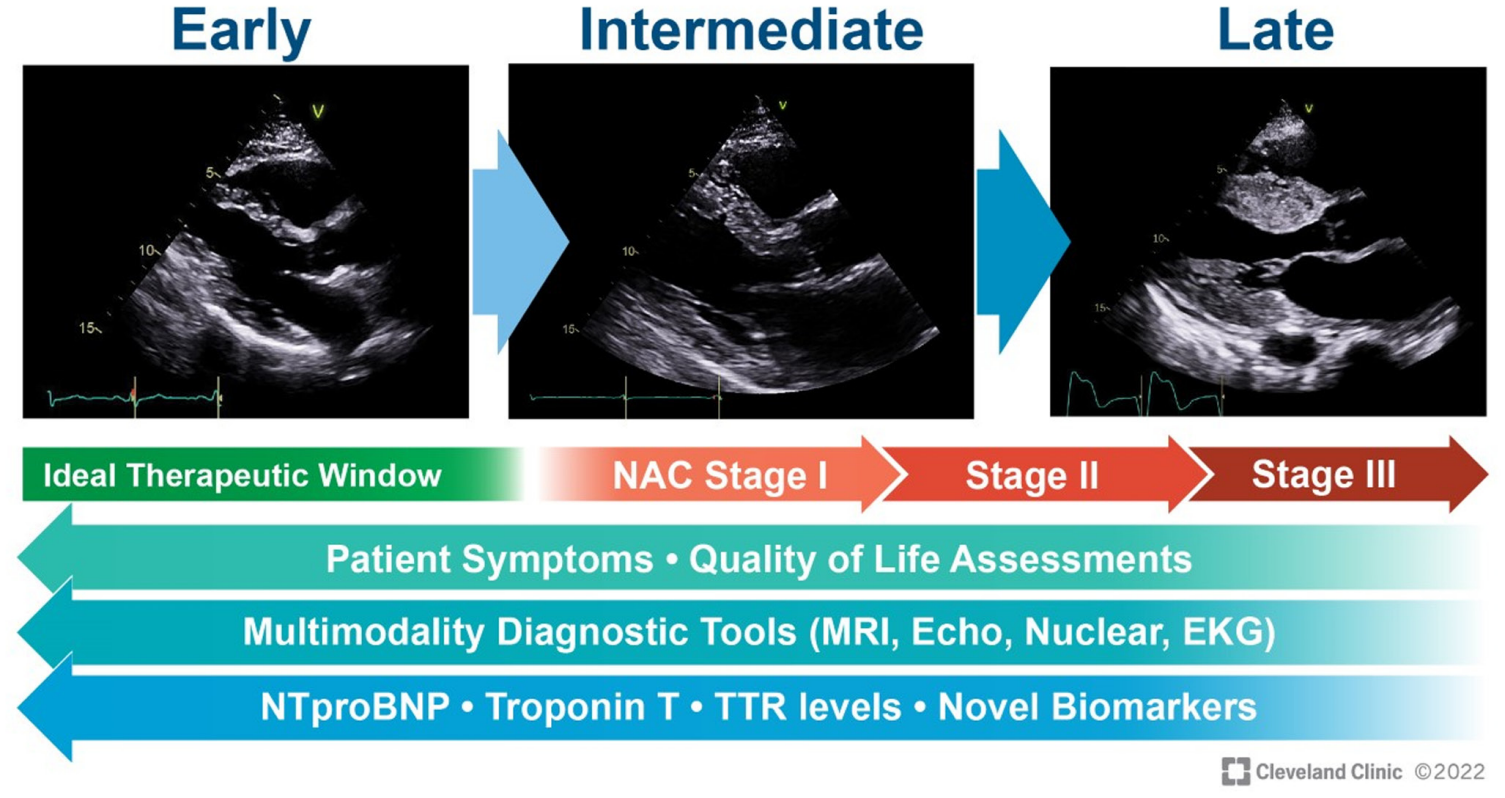
Representation of echocardiographic images (parasternal long-axis view) at different levels of wall thickness in transthyretin amyloid cardiomyopathy. The ideal therapeutic window would be to identify these patients at the earliest stage possible with near normal or mildly increased wall thickness before decline in quality of life, elevation in cardiac biomarkers, and overt cardiac manifestations of disease.^15^ NAC: National Amyloidosis Center; MRI: magnetic resonance imaging; Echo: echocardiography; EKG: electrocardiogram; NTproBNP: N-terminal-pro hormone brain natriuretic peptide; TTR: transthyretin

Contemporary data from a large referral center in the post-tafamidis period suggests that NAC staging is still highly prognostic in patients on transthyretin stabilizers; however, a large proportion of patients are still significantly advanced in their disease presentation, with 56% of patients with ATTR-CM being diagnosed with NAC stage II or III disease (Figure 3).^45^ Despite a rapid increase in the volume of cardiac scintigraphy performed at amyloidosis centers, the median time from symptom onset to diagnosis of ATTRwt-CM has not changed in large registry studies.^36,46,47^

Early diagnosis of ATTR-CM is paramount in preventing undue morbidity and mortality from the continued deposition of amyloid fibrils. The ATTR-ACT study showed that patients with New York Heart Association (NYHA) class I and II derived the most benefit of treatment when compared with NYHA III.^19^ Given that current therapeutics do not address existing deposition, misdiagnosis and delays result in potentially irreversible damage both in the heart and systemically.^2,27^

## Part IV: Opportunities for Earlier Identification of Cardiac Amyloidosis

### Tissue Screening

Because carpal tunnel syndrome and lumbar spinal stenosis have been associated with amyloid deposition in patients who later develop ATTR-CM, investigators have sought to understand the yield of active ascertainment strategies, which involves evaluating surgical pathology for the presence of amyloid fibrils.^29^

### Carpal Tunnel

**Figure 4:**
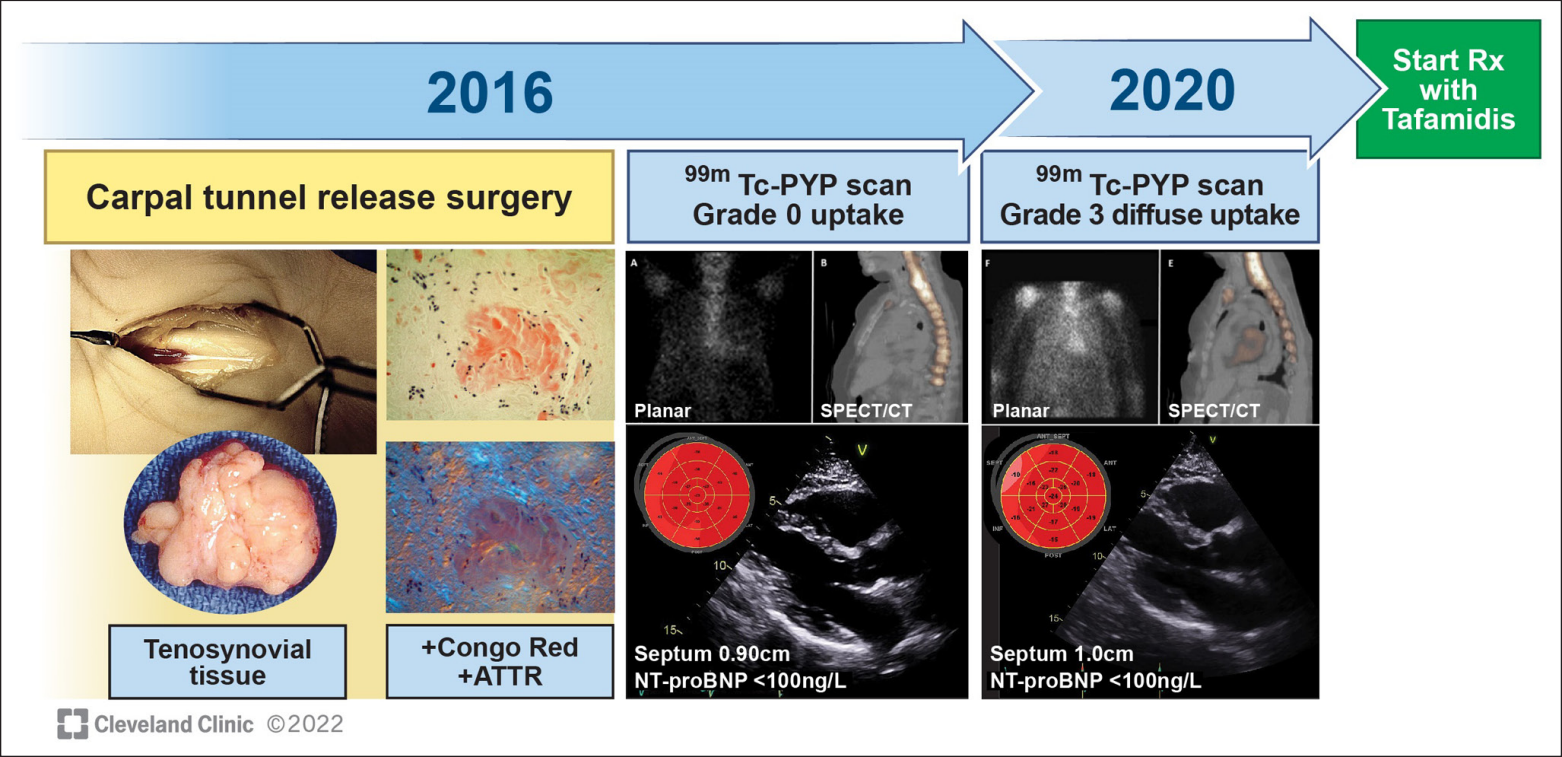
A case of early diagnosis and initiation of treatment in asymptomatic transthyretin (TTR) amyloid cardiomyopathy (ATTR-CM) through proactive tissue screening. A 73-year-old White female underwent carpal tunnel release surgery; Congo red staining of removed tenosynovial tissue was amyloid (+), typed as ATTR by mass spectrometry. TTR genetic testing revealed Ala 81Thr variant (+). At baseline, echocardiography with strain, N-terminal pro-brain natriuretic peptide (NT-proBNP), and ^99m^Tc-PYP scan showed no evidence of cardiac amyloid. At 4-year follow-up (age 77), the patient remained asymptomatic, with minimal to no change on echo and NT-proBNP, but she developed grade 3 diffuse uptake on ^99m^Tc-PYP scan. After shared decision making, the patient was started on tafamidis.

**Figure 5:**
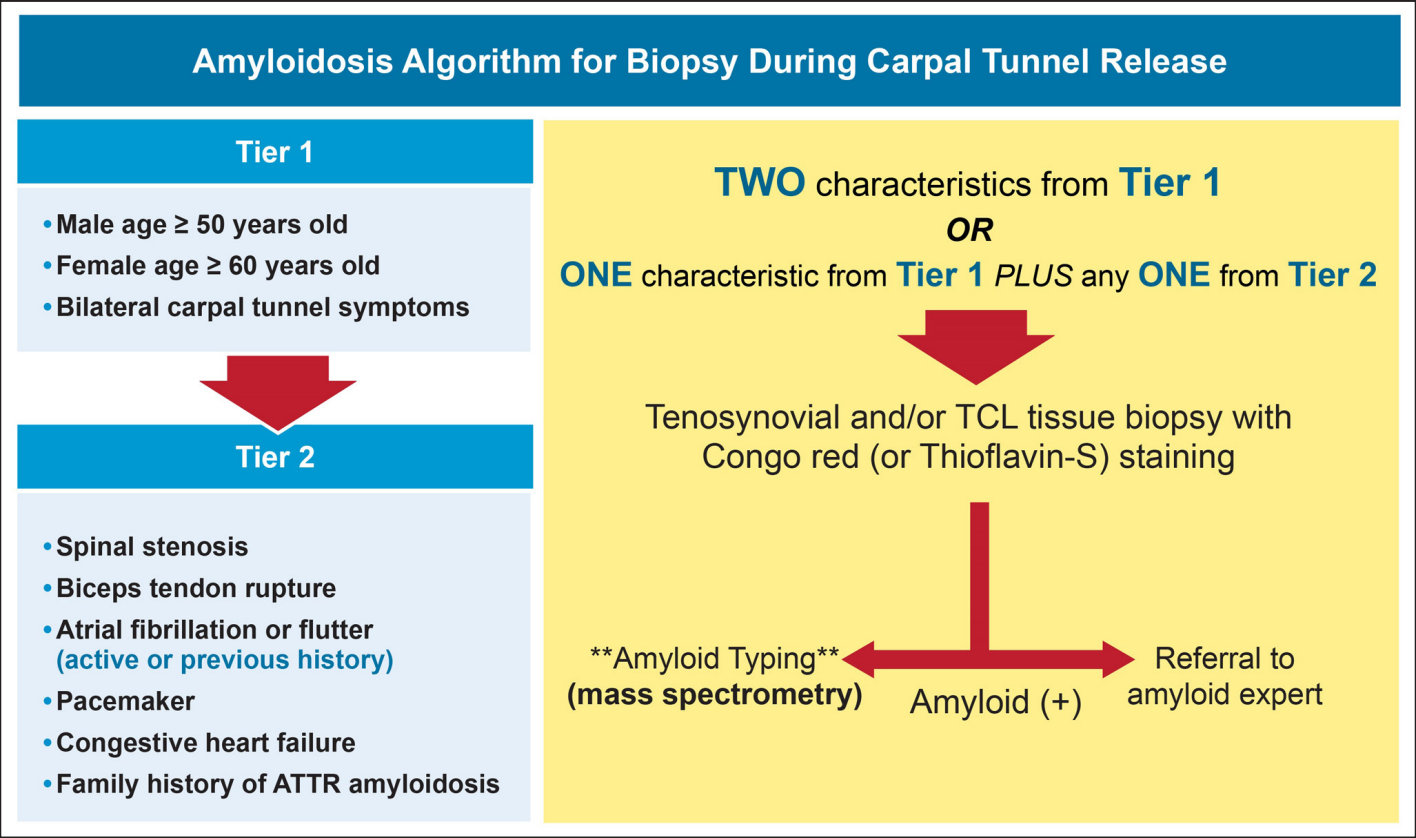
Cleveland Clinic institutional algorithm for tenosynovial biopsy during carpal tunnel release surgery utilizing a two-tier approach to select patients for prospective Congo red staining. If positive, further amyloid typing with mass spectrometry is performed and a referral is made to an amyloid specialist.^29^ TCL: transverse carpal ligament

In a prospective study of patients (males age ≥ 50 yrs, females age ≥ 60 yrs) undergoing carpal tunnel release surgery, Congo red staining of tenosynovial tissue detected amyloid deposits in 10.2% (10/98) of patients. After evaluation of the 10 biopsy positive patients (7 ATTR, 2 AL, and 1 untyped), it was found that 2 patients (1 AL and 1 ATTR) had cardiac involvement.^29,48^ One patient with TTR amyloid deposits in the tenosynovium developed asymptomatic subclinical ATTR-CM at 4-year follow-up (Figure 4).^49^ Currently, our institution is following the screening protocol that was used in the aforementioned study and has thus far found more than 100 tenosynovial samples positive for amyloid (unpublished data) (Figure 5). At the time of surgery, the yield for finding cardiac involvement is low; however, recent data suggest that longitudinal surveillance of these patients (particularly age > 70 years) may have significant yield.^50^

### Spinal Stenosis

Although surgical pathologic data has been mixed, 324 patients in one study underwent surgery for spinal stenosis, and 43 (13%) had ATTRwt in the ligamentum flavum, with 2 cases of confirmed cardiac involvement.^31,51,52^ In a recent publication, 34% of patients undergoing spinal stenosis surgery had amyloid deposition. Unfortunately, of those with amyloid deposition, more than one-third could not be accurately typed.^53^ Though sampling and analysis of surgical tissue at the time of surgical procedures may be a low-risk opportunity to identify patients who are at risk for developing ATTR-CM, how to best define the surgical screening cohort for the highest-risk patients, as well as the optimal plan for surveillance of patients with amyloid deposition, merits further investigation.

### High-Risk Elderly Subpopulations

Other subpopulations considered at high pre-test risk include outpatients and inpatients presenting with heart failure and elderly patients referred for transcatheter aortic valve replacement (TAVR).^12,13,54^

### Heart Failure with Preserved Ejection Fraction

One study prospectively screened consecutive patients ≥ 60 years old admitted with acute HFpEF and LV wall thickness ≥ 12 mm. Of the 120 patients, 16 (13.3%) had cardiac scintigraphy (99mTc-DPD) consistent with CA, and all were ATTRwt.^13^ There was no significant difference in the proportion of men and women who had ATTRwt-CM, raising the possibility of ascertainment bias in registries that have consistently shown a dramatic predominance of males.^55^ Additionally, in patients presenting to a dedicated HFpEF clinic, 14% of consecutive patients who underwent endomyocardial biopsy were found to have CA.^12^ Pathways to identify and screen inpatients and ambulatory HF patients with risk factors for ATTR-CM may be a high-yield opportunity.

Lastly, given the heterogeneity of HFpEF, there is mounting evidence that a significant proportion of amyloid patients may have been inadvertently enrolled in prior HFpEF clinical trials.^56^ Some have proposed implementing “red-flag” screening and evaluation prior to enrolling patients in HFpEF trials to prevent off-target use of heart failure therapeutics.^57^

### Transcatheter Aortic Valve Replacement

Severe calcific aortic stenosis (AS) represents an elderly population for which targeted screening has had variable but significant yield.^54,58^ Patients referred for TAVR at three international sites underwent cardiac scintigraphy and 11.8% (48/407) were scan positive. The authors developed a clinical risk score utilizing LV remodeling (hypertrophy/diastolic dysfunction), age, injury (high-sensitivity troponin T), and electrical abnormalities (right bundle branch block/low voltages) with reasonable sensitivity and specificity for the diagnosis of AS with concomitant ATTR-CM.^54^

Another study found a slightly higher prevalence of ATTR-CM (13%) in an older population referred for TAVR.^59^ Again, patient characteristics including thicker LV walls, higher NT-proBNP, and lower ECG voltage were present in a higher proportion of patients with ATTR-CM compared with non-ATTR-CM AS. Outcomes did not differ between groups in the latter study over a median 19-month follow-up period. Importantly, modified screening criteria will be needed as the population referred for TAVR continues to evolve.

### Black Patients

Given the prevalence of the V122I variant (3% to 4%) in the self-identified Black population in the United States, there may be significant opportunity to proactively identify early heart failure phenotypes through imaging, biomarker-based, and genetic screening for ATTRv-CM.^60^ In one study, having the V122I variant was associated with increased risk of incident heart failure, heart failure hospitalization, and chronic myocardial injury manifested by elevation in cardiac troponin compared with control patients.^61^ Community-based efforts aimed at earlier identification of ATTRv-CM in Black patients is an area that merits further exploration.^61,62^ Studies such as SCAN-MP (Screening for Cardiac Amyloidosis Using Nuclear Imaging for Minority Populations; NCT 03812172), a prospective cohort study using bone scintigraphy recruiting Black and Hispanic patients with heart failure, will aim to detect ATTR-CM in Black patients regardless of genotype.^63^

### Electronic Health Records-based Screening Cohorts

Given the broad use of EHR, the ability to identify patients with risk factors for ATTR-CM using diagnosis codes and imaging reports has emerged as a powerful tool to reduce the diagnostic inertia associated with ATTR-CM. In an illustrative population-based study conducted through an integrated EHR-derived cohort in the United States, patients with a validated HF diagnosis, age ≥ 60 years, ejection fraction ≥ 40%, and ventricular wall thickness ≥ 12 mm were assessed for ATTR-CM.^64^ In 1,235 patients without screening outreach, 1.3% were diagnosed with ATTR-CM. Meanwhile, in the proactive screening cohort of 286 patients who consented to cardiac scintigraphy, 6.3% were diagnosed with ATTR-CM.^64^ Importantly, EHR-facilitated clinical suspicion for CA can expand the diagnostic pathway beyond the tertiary referral center.

### Learning Algorithms

Machine learning may be additive to traditional EHR-based cohorts, offering a novel approach to identifying patients with high pre-test risk of ATTR-CM. A recent study from Huda et al. used a large claims dataset to develop a cohort of patients with heart failure with ATTR-CM and non-ATTR-CM HF serving as the control population.^65^ International Classification of Diseases tenth revision codes were used to train a random forest machine learning model, which was validated with multiple external cohorts. Various combinations of cardiac and noncardiac diagnoses that demonstrated higher prevalence in ATTR-CM compared with non-ATTR-CM controls were described. For example, the combination of AF, joint disorders, and HFpEF was 29.7% prevalent in ATTR-CM compared with 7.0% in non-amyloid HF. The authors envision future studies that prospectively validate their modeling by utilizing automated laboratory testing, echocardiography (with global longitudinal strain), and cardiac scintigraphy in patients at risk for ATTR-CM.^65^

Learning algorithms also may aid the expansion of imaging-based surveillance beyond the formal clinic/hospital setting and enable earlier disease detection. There is pilot data to suggest that imaging algorithms can enable novices without experience in ultrasonography to obtain basic diagnostic transthoracic echocardiographic images. This could facilitate a future state wherein targeted community-based screening could be scaled using algorithmic image acquisition as well as interpretation.^66,67^ Lastly, artificial intelligence (AI) may enhance our ability to detect CA earlier through AI modeling that predicts early disease, utilizing the combination of electrocardiographic and echocardiographic data as inputs.^68,69^

### Genetic TTR Variants

Patient education, genetic counseling, and patient advocacy could increase the identification of TTR variants in the first-degree relatives of patients with known disease. Furthermore, gene biobanks linked to EHR have emerged as effective tools for the characterization of rare genetic variants.^70^ EHR-linked biobanks have recently been leveraged to track associations between the TTR V122I variant and heart failure as well as polyneuropathy in patients with African or Hispanic/Latino ancestry.^71,72^ Given that serial imaging and biomarkers, particularly in patients with a known *TTR* gene variant, can diagnose presymptomatic disease, the use of EHR-linked biobanks to better understand natural history and facilitate early disease intervention has significant potential.^49,70^

Once a patient with a TTR variant is identified, how to surveil for phenotypic expression remains unclear. However, it should be tailored to the specific variant’s organ tropism and the age at which the proband developed disease. Recommended baseline cardiac and neurologic testing repeated at various intervals should be done in consultation with amyloidosis experts.

Innovative work in determining whether circulating non-native TTR levels (which are breakdown products of the TTR tetramer) correlate with clinical status and treatment response in patients with ATTRv amyloidosis is currently underway and has shown promising mechanistic potential.^73^ The Scripps Research Institute has developed a peptide-based probe that specifically labels and quantifies non-native TTR levels in the blood (TTR oligomers). The elevation of these TTR oligomers can suggest that the TTR misfolding process is active and might predate the subclinical or clinical phenotypic expression of ATTR amyloidosis. The “Monitoring of Early Disease Progression in Hereditary Transthyretin Amyloidosis” (MED-hATTR) Study is currently testing the longitudinal monitoring of these non-native TTR levels in asymptomatic carriers of TTR variants to predict the development of early disease.^74^

## Part V: Conclusions and Future Directions

**Figure 6:**
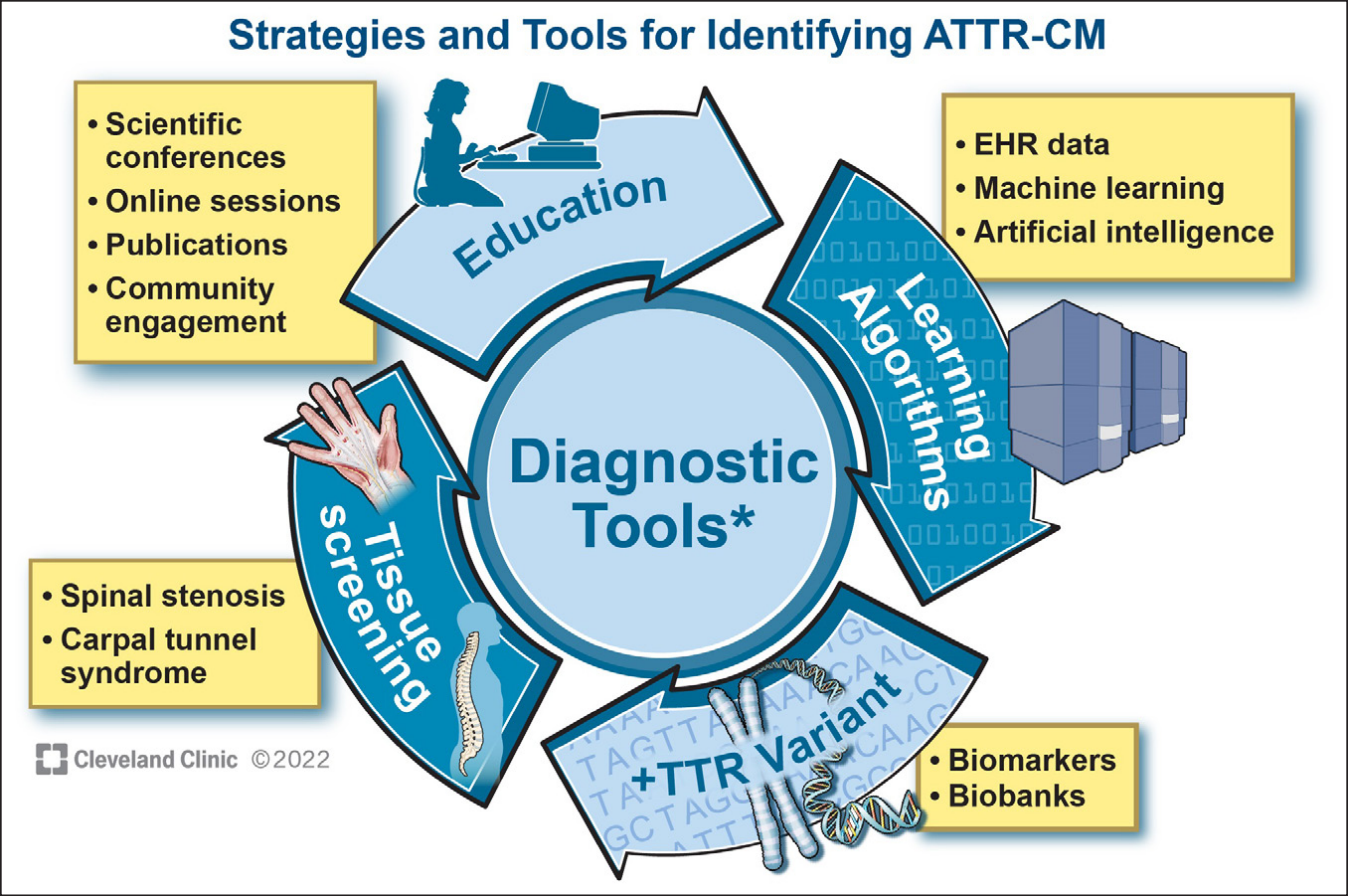
Strategies and tools to detect asymptomatic transthyretin (TTR) amyloid cardiomyopathy (ATTR-CM) earlier in the disease course. Patient and clinician engagement/education, tissue screening, biomarkers and gene biobanks, and analyses of clinical data derived from the electronic health records, longitudinal registries, and clinical trials can be used to calibrate the optimal use of diagnostic tools (ie, targeted questionnaires, electrocardiogram, echocardiography, cardiac magnetic resonance imaging, cardiac scintigraphy, cardiac biomarkers). Over time, learning algorithms using machine learning and artificial intelligence may allow for the iterative improvement of accurate diagnosis and enable patient identification and appropriate screening of patients at risk for ATTR-CM. EHR: electronic health record

Despite the rapid expansion of non-biopsy diagnostic testing, contemporary patients still present with advanced disease.^8,47,75^ Therapy for ATTR-CM is most effective when administered before significant symptoms of cardiac dysfunction manifest; therefore, early identification of affected individuals is paramount.^7^ Community engagement and ensuring that a broad range of clinicians have working knowledge of how to screen for ATTR-CM in everyday practice will be an important step in moving disease identification further upstream (Figure 6). However, reliance on the appropriate and timely diagnosis by individual clinicians may continue to underperform. Systems of care that operationalize screening of high-risk subpopulations and prospective validation of EHR-based approaches to ATTR-CM identification are needed. The ideal yield of screening efforts has yet to be determined, and further research may elucidate the most practical and effective approaches. In the meantime, maintaining a high index of suspicion and having a low threshold for comprehensive screening for ATTR-CM will best serve our patients.

## Key Points

- Contemporary patients with cardiac amyloidosis too often present with advanced disease.
- Therapeutics for transthyretin cardiomyopathy (ATTR-CM) are most effective when administered before significant symptoms of cardiac dysfunction manifest, making early identification of affected individuals essential.
- Targeted screening of special populations may facilitate earlier diagnosis.
- Systems of care that operationalize screening of high-risk subpopulations and prospective validation of novel approaches to ATTR-CM identification are needed.

## References

[1] Garcia-Pavia P, Rapezzi C, Adler Y, et al. Diagnosis and treatment of cardiac amyloidosis: a position statement of the ESC Working Group on Myocardial and Pericardial Diseases. Eur Heart J. 2021 Apr 21;42(16):1554-1568. doi: 10.1093/eurheartj/ehab072 33825853 PMC8060056

[2] Maurer MS, Elliott P, Comenzo R, Semigran M, Rapezzi C. Addressing Common Questions Encountered in the Diagnosis and Management of Cardiac Amyloidosis. Circulation. 2017 Apr 4;135(14):1357-1377. doi: 10.1161/CIRCULATIONAHA.116.024438 28373528 PMC5392416

[3] Donnelly JP, Hanna M. Cardiac amyloidosis: An update on diagnosis and treatment. Cleve Clin J Med. 2017 Dec;84(12 Suppl 3):12-26. doi: 10.3949/ccjm.84.s3.02 29257735

[4] Witteles RM, Bokhari S, Damy T, et al. Screening for Transthyretin Amyloid Cardiomyopathy in Everyday Practice. JACC Heart Fail. 2019 Aug;7(8):709-716. doi: 10.1016/j.jchf.2019.04.010 31302046

[5] Bianchi G, Zhang Y, Comenzo RL. AL Amyloidosis: Current Chemotherapy and Immune Therapy Treatment Strategies: JACC: CardioOncology State-of-the-Art Review. JACC CardioOncol. 2021 Oct 19;3(4):467-487. doi: 10.1016/j.jaccao.2021.09.003 34729520 PMC8543128

[6] Maurer MS, Bokhari S, Damy T, et al. Expert Consensus Recommendations for the Suspicion and Diagnosis of Transthyretin Cardiac Amyloidosis. Circ Heart Fail. 2019 Sep;12(9):e006075. doi: 10.1161/CIRCHEARTFAILURE.119.006075 31480867 PMC6736650

[7] Kittleson MM, Maurer MS, Ambardekar AV, et al. Cardiac Amyloidosis: Evolving Diagnosis and Management: A Scientific Statement From the American Heart Association. Circulation. 2020 Jul 7;142(1):e7-e22. doi: 10.1161/CIR.0000000000000792 32476490

[8] Lane T, Fontana M, Martinez-Naharro A, et al. Natural History, Quality of Life, and Outcome in Cardiac Transthyretin Amyloidosis. Circulation. 2019 Jul 2;140(1):16-26. doi: 10.1161/CIRCULATIONAHA.118.038169 31109193

[9] Gillmore JD, Damy T, Fontana M, et al. A new staging system for cardiac transthyretin amyloidosis. Eur Heart J. 2018 Aug 7;39(30):2799-2806. doi: 10.1093/eurheartj/ehx589 29048471

[10] Grogan M, Scott CG, Kyle RA, et al. Natural History of Wild-Type Transthyretin Cardiac Amyloidosis and Risk Stratification Using a Novel Staging System. J Am Coll Cardiol. 2016 Sep 6;68(10):1014-20. doi: 10.1016/j.jacc.2016.06.033 27585505

[11] Ruberg FL, Grogan M, Hanna M, Kelly JW, Maurer MS. Transthyretin Amyloid Cardiomyopathy: JACC State-of-the-Art Review. J Am Coll Cardiol. 2019 Jun 11;73(22):2872-2891. doi: 10.1016/j.jacc.2019.04.003 31171094 PMC6724183

[12] Hahn VS, Yanek LR, Vaishnav J, et al. Endomyocardial Biopsy Characterization of Heart Failure With Preserved Ejection Fraction and Prevalence of Cardiac Amyloidosis. JACC Heart Fail. 2020 Sep;8(9):712-724. doi: 10.1016/j.jchf.2020.04.007 32653448 PMC7604801

[13] González-López E, Gallego-Delgado M, Guzzo-Merello G, et al. Wild-type transthyretin amyloidosis as a cause of heart failure with preserved ejection fraction. Eur Heart J. 2015 Oct 7;36(38):2585-94. doi: 10.1093/eurheartj/ehv338 26224076

[14] Tanskanen M, Peuralinna T, Polvikoski T, et al. Senile systemic amyloidosis affects 25% of the very aged and associates with genetic variation in alpha2-macroglobulin and tau: a population-based autopsy study. Ann Med. 2008;40(3):232-9. doi: 10.1080/07853890701842988 18382889

[15] Grodin JL, Maurer MS. The Truth Is Unfolding About Transthyretin Cardiac Amyloidosis. Circulation. 2019 Jul 2;140(1):27-30. doi: 10.1161/CIRCULATIONAHA.119.041015 31549879 PMC6761830

[16] Stern LK, Patel J. Cardiac Amyloidosis Treatment. Methodist Debakey Cardiovasc J. 2022 Mar 14;18(2):59-72. doi: 10.14797/mdcvj.1050 PMC8932359 35414852

[17] Donnellan E, Wazni OM, Hanna M, et al. Atrial Fibrillation in Transthyretin Cardiac Amyloidosis: Predictors, Prevalence, and Efficacy of Rhythm Control Strategies. JACC Clin Electrophysiol. 2020 Sep;6(9):1118-1127. doi: 10.1016/j.jacep.2020.04.019 32972546

[18] Damy T, Maurer MS, Rapezzi C, et al. Clinical, ECG and echocardiographic clues to the diagnosis of TTR-related cardiomyopathy. Open Heart. 2016 Feb 8;3(1):e000289. doi: 10.1136/openhrt-2015-000289 26870387 PMC4746524

[19] Maurer MS, Schwartz JH, Gundapaneni B, et al. Tafamidis Treatment for Patients with Transthyretin Amyloid Cardiomyopathy. N Engl J Med. 2018 Sep 13;379(11):1007-1016. doi: 10.1056/NEJMoa1805689 30145929

[20] Damy T, Kristen AV, Suhr OB, et al. Transthyretin cardiac amyloidosis in continental Western Europe: an insight through the Transthyretin Amyloidosis Outcomes Survey (THAOS). Eur Heart J. 2019 Apr 1;43(5):391-400. doi: 10.1093/eurheartj/ehz173 30938420 PMC8825236

[21] Siqueira-Filho AG, Cunha CL, Tajik AJ, Seward JB, Schattenberg TT, Giuliani ER. M-mode and two-dimensional echocardiographic features in cardiac amyloidosis. Circulation. 1981 Jan;63(1):188-96. doi: 10.1161/01.cir.63.1.188 7438392

[22] Martyn T, Saef J, Dey AR, et al. Racial and Genetic Differences in Presentation of Transthyretin Amyloid Cardiomyopathy With Impaired Left Ventricular Function. JACC Heart Fail. 2022 Sep;10(9):689-691. doi: 10.1016/j.jchf.2022.06.006 36049818

[23] Phelan D, Collier P, Thavendiranathan P, et al. Relative apical sparing of longitudinal strain using two-dimensional speckle-tracking echocardiography is both sensitive and specific for the diagnosis of cardiac amyloidosis. Heart. 2012 Oct;98(19):1442-8. doi: 10.1136/heartjnl-2012-302353 22865865

[24] Siddiqi OK, Ruberg FL. Cardiac amyloidosis: An update on pathophysiology, diagnosis, and treatment. Trends Cardiovasc Med. 2018 Jan;28(1):10-21. doi: 10.1016/j.tcm.2017.07.004 28739313 PMC5741539

[25] Donnellan E, Wazni O, Kanj M, et al. Atrial fibrillation ablation in patients with transthyretin cardiac amyloidosis. Europace. 2020 Feb 1;22(2):259-264. doi: 10.1093/europace/euz314 32031230

[26] Bukhari S, Barakat AF, Eisele YS, et al. Prevalence of Atrial Fibrillation and Thromboembolic Risk in Wild-Type Transthyretin Amyloid Cardiomyopathy. Circulation. 2021 Mar 30;143(13):1335-1337. doi: 10.1161/CIRCULATIONAHA.120.052136 33779268

[27] González-López E, López-Sainz Á, Garcia-Pavia P. Diagnosis and Treatment of Transthyretin Cardiac Amyloidosis. Progress and Hope. Rev Esp Cardiol (Engl Ed). 2017 Nov;70(11):991-1004. doi: 10.1016/j.rec.2017.05.036 28870641

[28] Huang J, Zhao S, Chen Z, Zhang S, Lu M. Contribution of Electrocardiogram in the Differentiation of Cardiac Amyloidosis and Nonobstructive Hypertrophic Cardiomyopathy. Int Heart J. 2015;56(5):522-6. doi: 10.1536/ihj.15-005 26346516

[29] Sperry BW, Reyes BA, Ikram A, et al. Tenosynovial and Cardiac Amyloidosis in Patients Undergoing Carpal Tunnel Release. J Am Coll Cardiol. 2018 Oct 23;72(17):2040-2050. doi: 10.1016/j.jacc.2018.07.092 30336828

[30] Geller HI, Singh A, Alexander KM, Mirto TM, Falk RH. Association Between Ruptured Distal Biceps Tendon and Wild-Type Transthyretin Cardiac Amyloidosis. JAMA. 2017 Sep 12;318(10):962-963. doi: 10.1001/jama.2017.9236 28898370 PMC5818850

[31] Westermark P, Westermark GT, Suhr OB, Berg S. Transthyretin-derived amyloidosis: probably a common cause of lumbar spinal stenosis. Ups J Med Sci. 2014 Aug;119(3):223-8. doi: 10.3109/03009734.2014.895786 24620715 PMC4116761

[32] Stein K, Störkel S, Linke RP, Goebel HH. Chemical heterogeneity of amyloid in the carpal tunnel syndrome. Virchows Arch A Pathol Anat Histopathol. 1987;412(1):37-45. doi: 10.1007/BF00750729 3120402

[33] Karam C, Dimitrova D, Christ M, Heitner SB. Carpal tunnel syndrome and associated symptoms as first manifestation of hATTR amyloidosis. Neurol Clin Pract. 2019 Aug;9(4):309-313. doi: 10.1212/CPJ.0000000000000640 31583185 PMC6745748

[34] Sperry BW, Khedraki R, Gabrovsek A, et al. Cardiac Amyloidosis Screening at Trigger Finger Release Surgery. Am J Cardiol. 2021 Dec 1;160:96-98. doi: 10.1016/j.amjcard.2021.08.049 34620488

[35] Nakagawa M, Sekijima Y, Yazaki M, et al. Carpal tunnel syndrome: a common initial symptom of systemic wild-type ATTR (ATTRwt) amyloidosis. Amyloid. 2016;23(1):58-63. doi: 10.3109/13506129.2015.1135792 26852880

[36] Hanna M, Ruberg FL, Maurer MS, et al. Cardiac Scintigraphy With Technetium-99m-Labeled Bone-Seeking Tracers for Suspected Amyloidosis: JACC Review Topic of the Week. J Am Coll Cardiol. 2020 Jun 9;75(22):2851-2862. doi: 10.1016/j.jacc.2020.04.022 32498813

[37] Marume K, Takashio S, Nishi M, et al. Combination of Commonly Examined Parameters Is a Useful Predictor of Positive 99 mTc-Labeled Pyrophosphate Scintigraphy Findings in Elderly Patients With Suspected Transthyretin Cardiac Amyloidosis. Circ J. 2019 Jul 25;83(8):1698-1708. doi: 10.1253/circj.CJ-19-0255 31189791

[38] Davies DR, Redfield MM, Scott CG, et al. A Simple Score to Identify Increased Risk of Transthyretin Amyloid Cardiomyopathy in Heart Failure With Preserved Ejection Fraction. JAMA Cardiol. 2022 Sep 7;e221781. doi: 10.1001/jamacardio.2022.1781 PMC9453635 36069809

[39] Dorbala S, Ando Y, Bokhari S, et al. ASNC/AHA/ASE/EANM/HFSA/ISA/SCMR/SNMMI Expert Consensus Recommendations for Multimodality Imaging in Cardiac Amyloidosis: Part 1 of 2-Evidence Base and Standardized Methods of Imaging. J Card Fail. 2019 Nov;25(11):e1-e39. doi: 10.1016/j.cardfail.2019.08.001 31473268

[40] Dorbala S, Ando Y, Bokhari S, et al. ASNC/AHA/ASE/EANM/HFSA/ISA/SCMR/SNMMI Expert Consensus Recommendations for Multimodality Imaging in Cardiac Amyloidosis: Part 2 of 2-Diagnostic Criteria and Appropriate Utilization. J Card Fail. 2019 Nov;25(11):854-865. doi: 10.1016/j.cardfail.2019.08.002 31473267

[41] Rapezzi C, Elliott P, Damy T, et al. Efficacy of Tafamidis in Patients With Hereditary and Wild-Type Transthyretin Amyloid Cardiomyopathy: Further Analyses From ATTR-ACT. JACC Heart Fail. 2021 Feb;9(2):115-123. doi: 10.1016/j.jchf.2020.09.011 33309574

[42] Adams D, Gonzalez-Duarte A, O’Riordan WD, et al. Patisiran, an RNAi Therapeutic, for Hereditary Transthyretin Amyloidosis. N Engl J Med. 2018 Jul 5;379(1):11-21. doi: 10.1056/NEJMoa1716153 29972753

[43] Benson MD, Waddington-Cruz M, Berk JL, et al. Inotersen Treatment for Patients with Hereditary Transthyretin Amyloidosis. N Engl J Med. 2018 Jul 5;379(1):22-31. doi: 10.1056/NEJMoa1716793 29972757 PMC12611561

[44] Gillmore JD, Gane E, Taubel J, et al. CRISPR-Cas9 In Vivo Gene Editing for Transthyretin Amyloidosis. N Engl J Med. 2021 Aug 5;385(6):493-502. doi: 10.1056/NEJMoa2107454 34215024

[45] Martyn T, Saef J, Hussain M, et al. The Association of Cardiac Biomarkers, the Intensity of Tc99 Pyrophosphate Uptake, and Survival in Patients Evaluated for Transthyretin Cardiac Amyloidosis in the Early Therapeutics Era. J Card Fail. 2022 Jul 14;S1071-9164(22)00581-4. doi: 10.1016/j.cardfail.2022.06.005 35843490

[46] Nativi-Nicolau J, Siu A, Dispenzieri A, et al. Temporal Trends of Wild-Type Transthyretin Amyloid Cardiomyopathy in the Transthyretin Amyloidosis Outcomes Survey. JACC CardioOncol. 2021 Oct 19;3(4):537-546. doi: 10.1016/j.jaccao.2021.08.009 34729526 PMC8543133

[47] Poterucha TJ, Elias P, Bokhari S, et al. Diagnosing Transthyretin Cardiac Amyloidosis by Technetium Tc 99m Pyrophosphate: A Test in Evolution. JACC Cardiovasc Imaging. 2021 Jun;14(6):1221-1231. doi: 10.1016/j.jcmg.2020.08.027 33221204 PMC8113330

[48] Donnelly JP, Hanna M, Sperry BW, Seitz WH Jr. Carpal Tunnel Syndrome: A Potential Early, Red-Flag Sign of Amyloidosis. J Hand Surg Am. 2019 Oct;44(10):868-876. doi: 10.1016/j.jhsa.2019.06.016 31400950

[49] Hussain M, Sperry BW, Hanna M, Jaber WA. Conversion of 99mtechnetium-pyrophosphate scintigraphy in a patient with hereditary ATTR amyloidosis: importance of repeat scanning. Eur Heart J Case Rep. 2020 Nov 9;4(6):1-2. doi: 10.1093/ehjcr/ytaa386 PMC7793235 33447723

[50] Westin O, Fosbøl EL, Maurer MS, et al. Screening for Cardiac Amyloidosis 5 to 15 Years After Surgery for Bilateral Carpal Tunnel Syndrome. J Am Coll Cardiol. 2022 Sep 6;80(10): 967-977. doi: 10.1016/j.jacc.2022.06.026 36049804

[51] Godara A, Riesenburger RI, Zhang DX, et al. Association between spinal stenosis and wild-type ATTR amyloidosis. Amyloid. 2021 Dec;28(4):226-233. doi: 10.1080/13506129.2021.1950681 34263670

[52] Yanagisawa A, Ueda M, Sueyoshi T, et al. Amyloid deposits derived from transthyretin in the ligamentum flavum as related to lumbar spinal canal stenosis. Mod Pathol. 2015 Feb;28(2):201-7. doi: 10.1038/modpathol.2014.102 25189643

[53] Maurer MS, Smiley D, Simsolo E, et al. Analysis of lumbar spine stenosis specimens for identification of amyloid. J Am Geriatr Soc. 2022 Aug 5. doi: 10.1111/jgs.17976 PMC9771886 35929177

[54] Nitsche C, Scully PR, Patel KP, et al. Prevalence and Outcomes of Concomitant Aortic Stenosis and Cardiac Amyloidosis. J Am Coll Cardiol. 2021 Jan 19;77(2):128-139. doi: 10.1016/j.jacc.2020.11.006 33181246 PMC7805267

[55] DeFilippis EM, Beale A, Martyn T, et al. Heart Failure Subtypes and Cardiomyopathies in Women. Circ Res. 2022 Feb 18;130(4):436-454. doi: 10.1161/CIRCRESAHA.121.319900 35175847 PMC10361647

[56] Sperry BW, Hanna M, Shah SJ, Jaber WA, Spertus JA. Spironolactone in Patients With an Echocardiographic HFpEF Phenotype Suggestive of Cardiac Amyloidosis: Results From TOPCAT. JACC Heart Fail. 2021 Nov;9(11):795-802. doi: 10.1016/j.jchf.2021.06.007 34509404

[57] Oghina S, Bougouin W, Bézard M, et al. The Impact of Patients With Cardiac Amyloidosis in HFpEF Trials. JACC Heart Fail. 2021 Mar;9(3):169-178. doi: 10.1016/j.jchf.2020.12.005 33549560

[58] Castaño A, Narotsky DL, Hamid N, et al. Unveiling transthyretin cardiac amyloidosis and its predictors among elderly patients with severe aortic stenosis undergoing transcatheter aortic valve replacement. Eur Heart J. 2017 Oct 7;38(38):2879-2887. doi: 10.1093/eurheartj/ehx350 29019612 PMC5837725

[59] Scully PR, Patel KP, Treibel TA, et al. Prevalence and outcome of dual aortic stenosis and cardiac amyloid pathology in patients referred for transcatheter aortic valve implantation. Eur Heart J. 2020 Aug 1;41(29):2759-2767. doi: 10.1093/eurheartj/ehaa170 32267922 PMC7395329

[60] Quarta CC, Buxbaum JN, Shah AM, et al. The amyloidogenic V122I transthyretin variant in elderly black Americans. N Engl J Med. 2015 Jan 1;372(1):21-9. doi: 10.1056/NEJMoa1404852 25551524 PMC4382209

[61] Coniglio AC, Segar MW, Loungani RS, et al. Transthyretin V142I Genetic Variant and Cardiac Remodeling, Injury, and Heart Failure Risk in Black Adults. JACC Heart Fail. 2022 Feb;10(2):129-138. doi: 10.1016/j.jchf.2021.09.006 35115086 PMC12707018

[62] Sinha A, Zheng Y, Nannini D, et al. Association of the V122I Transthyretin Amyloidosis Genetic Variant With Cardiac Structure and Function in Middle-aged Black Adults: Coronary Artery Risk Development in Young Adults (CARDIA) Study. JAMA Cardiol. 2020 Dec 23;6(6):1-5. doi: 10.1001/jamacardio.2020.6623 PMC7758832 33355618

[63] ClinicalTrials.gov [Internet]. Bethesda, MD: US National Library of Medicine; c2022. Screening for Cardiac Amyloidosis With Nuclear Imaging for Minority Populations (SCAN-MP); 2022 May 23 [cited 2022 Nov 17]. Available from: https://clinicaltrials.gov/ct2/show/NCT03812172

[64] AbouEzzeddine OF, Davies DR, Scott CG, et al. Prevalence of Transthyretin Amyloid Cardiomyopathy in Heart Failure With Preserved Ejection Fraction. JAMA Cardiol. 2021 Nov 1;6(11):1267-1274. doi: 10.1001/jamacardio.2021.3070 34431962 PMC8387947

[65] Huda A, Castaño A, Niyogi A, et al. A machine learning model for identifying patients at risk for wild-type transthyretin amyloid cardiomyopathy. Nat Commun. 2021 May 11;12(1):2725. doi: 10.1038/s41467-021-22876-9 33976166 PMC8113237

[66] Narang A, Bae R, Hong H, et al. Utility of a Deep-Learning Algorithm to Guide Novices to Acquire Echocardiograms for Limited Diagnostic Use. JAMA Cardiol. 2021 Jun 1;6(6):624-632. doi: 10.1001/jamacardio.2021.0185 33599681 PMC8204203

[67] Zhang J, Gajjala S, Agrawal P, et al. Fully Automated Echocardiogram Interpretation in Clinical Practice. Circulation. 2018 Oct 16;138(16):1623-1635. doi: 10.1161/CIRCULATIONAHA.118.034338 30354459 PMC6200386

[68] Grogan M, Lopez-Jimenez F, Cohen-Shelly M, et al. Artificial Intelligence-Enhanced Electrocardiogram for the Early Detection of Cardiac Amyloidosis. Mayo Clin Proc. 2021 Nov;96(11):2768-2778. doi: 10.1016/j.mayocp.2021.04.023 34218880

[69] Goto S, Mahara K, Beussink-Nelson L, et al. Artificial intelligence-enabled fully automated detection of cardiac amyloidosis using electrocardiograms and echocardiograms. Nat Commun. 2021 May 11;12(1):2726. doi: 10.1038/s41467-021-22877-8 33976142 PMC8113484

[70] Reza N, Damrauer SM. Toward Population-Based Genetic Screening for Hereditary Amyloidosis. JACC CardioOncol. 2021 Oct 19;3(4):562-564. doi: 10.1016/j.jaccao.2021.09.005 34729528 PMC8543136

[71] Parker MM, Damrauer SM, Tcheandjieu C, et al. Association of the transthyretin variant V122I with polyneuropathy among individuals of African ancestry. Sci Rep. 2021 Jun 2;11(1):11645. doi: 10.1038/s41598-021-91113-6 34079032 PMC8172853

[72] Damrauer SM, Chaudhary K, Cho JH, et al. Association of the V122I Hereditary Transthyretin Amyloidosis Genetic Variant With Heart Failure Among Individuals of African or Hispanic/Latino Ancestry. JAMA. 2019 Dec 10;322(22):2191-2202. doi: 10.1001/jama.2019.17935 31821430 PMC7081752

[73] Jiang X, Labaudinière R, Buxbaum JN, et al. A circulating, disease-specific, mechanism-linked biomarker for ATTR polyneuropathy diagnosis and response to therapy prediction. Proc Natl Acad Sci U S A. 2021 Mar 2;118(9):e2016072118. doi: 10.1073/pnas.2016072118 33597308 PMC7936353

[74] ClinicalTrials.gov [Internet]. Bethesda, MD: US National Library of Medicine; c2022. Monitorial of Early Disease Progression in hereditary Transthyretin Amyloidosis (MED-hATTR); 2022 Mar 28 [cited 2022 Nov 11]. Available from: https://www.clinicaltrials.gov/ct2/show/NCT03431896

[75] Martinez-Naharro A, Treibel TA, Abdel-Gadir A, et al. Magnetic Resonance in Transthyretin Cardiac Amyloidosis. J Am Coll Cardiol. 2017 Jul 25;70(4):466-477. doi: 10.1016/j.jacc.2017.05.053 28728692

